# Multicentric Adenocarcinomas in a Long-Segment of Barrett’s Esophagus

**DOI:** 10.4137/cmo.s287

**Published:** 2008-06-09

**Authors:** Stefan Hartl, Joerg-Ruediger Siewert, Joerg Theisen

**Affiliations:** Department of Surgery, Klinikum rechts der Isar, Technische Universitaet München, Ismaningerstr.22, 81675 Munich, Germany

## Abstract

This report describes a complicated course of a 58-year-old patient with multicentric Barrett’s carcinoma within a long-segment of Barrett metaplasia. After abdominal-thoracic resection of the cancer, with incomplete removal of the long-segment metaplastic lesion, invasive carcinoma was diagnosed in the remnant Barrett’s segment. Endoscopic mucosal resection was done, but Barrett’s mucosa was left in situ again. Recurrent tumor growth was diagnosed only few months later. Finally, transthoracic complete resection on the remnant Barrett’s segment was performed. Thus, our case demonstrates impressively the appearance of multicentric adenocarcinomas in Barrett’s esophagus and underlines the necessity of resection of the complete Barrett mucosa.

## Introduction

Barrett’s esophagus (BE) is an acquired disease of the esophagus. Normal distal stratified squamous epithelium is transformed into metaplastic intestinal type columnar epithelium. Since the 1970s, there has been a remarkable change in the epidemiology of esophageal malignancy (1). The incidence of esophageal adenocarcinoma rose approximately sixfold in the United States. It is discussed, that chronic gastroesophageal reflux disease (GERD) causes acute mucosal injury, cellular proliferation, and specialized columnar metaplasia (2). Ingredients of the gastric refluxate—acid and pepsin—cause mucosal injury. Bile acids, bile lysolecithin and pancreatic trypsin are suspected to have additional malignant influence. It is uncertain, how much time it takes for a transformation to Barrett’s esophagus—and further (3).

Our case report is that of a patient who developed recurrent esophageal adenocarcinoma during one year after partial esophagectomy for Barrett carcinoma with incomplete resection of the premalignant lesion.

## Patient History

In January 2006, a 58-year-old male was admitted to our hospital with intraepithelial low grade neoplasia in the remnant Barrett esophagus. The patient’s height was 174 cm, the weight 86 kg. He was a non-smoker and non-alcohol-consumer. Previously the patient has been suffering from hypertension. Heart and renal function were normal.

In February 2005, a moderate differentiated Barrett’s adenocarcinoma (G2) had been found, 3 cm in diameter. No angioinvasion was observed. The carcinoma was initially located in the distal esophagus within a 15 cm long-segment of specialized intestinal metaplasia, reaching 5 cm below the upper esophageal sphincter. No thorough endoscopic biopsy study was done prior to the initial operation. Therefore no information on other occult areas of dysplastic tissue was available at that time.

An abdomino-thoracic procedure was performed in September 2005. The primary pathohistological examination revealed an early carcinoma with no lymph node involvement and complete resection, pT1 pN0(0/13) M0 R0. The resection margin was free of tumor or dysplastic areas. For the reconstruction the entire stomach was pulled up into the chest and the anastomosis was performed in the mid esophagus. The entire segment of intestinal metaplasia was not removed at this time.

Due to persistent reflux symptoms follow-up endoscopy was performed in October 2005. Biopsies of a slightly elevated area just above the anastomosis within the remnant 3 cm Barrett’s mucosa showed high-grade intraepithelial neoplasia. Only this visible lesion was biopsied, no 4 quadrant biopsies were performed at this time. Endoscopic mucosectomy was done. Pathologically, this 0.9x0.7x0.5 cm superficial tissue showed Barrett’s mucosa, and centrally a well-differenciated adenocarcinoma (M1). Due to persistent reflux symptoms and need for Proton-Pump-Inhibitors the patient was referred to our hospital, a tertiary referral center for esophageal diseases.

The patient was admitted in January 2006. On endoscopy, remaining Barrett’s tissue was found with remnant intraepithelial neoplasia in the area of the mucosectomy. Four-quadrant biopsies showed low-grade and high-grade intraepithelial neoplasia in the remaining Barrrett’s mucosa. Tumor marker CA 72-4 was elevated (8.8 U/ml). X-rays, using radiopaque material, showed a free esophageal passage. There were no strictures nearby the gastro-esophageal anastomosis.

A re-thoracotomy was performed and the entire segment of intestinal metaplasia removed. In order to locate the proximal extent of the Barrett’s segment metal clips were placed endoscopically one day prior of the operation. The resected tissue, 6x6x0.8 cm in size, showed a well differentiated tubular adenocarcinoma, which was limited to the mucosa (rpT1m pN0 (0/8), R0, G1, diameter 2 mm) with adjacent areas of dysplastic tissue. Extended Barrett’s mucosa was found in the area of the original anastomosis. The clips were verified within the specimen.

Since initially the entire stomach was placed in the thoracic cavity a gastric tube could be created through the thoracotomy approach with a high intrathoracic esophago-gastric anastomosis in the posterior mediastinal area was set up. The patient remained stable further on, and was discharged from our hospital on day 16 postoperatively.

## Discussion

After initial diagnosis of Barrett’s adenocarcinoma, our patient’s esophagus was just partially removed, including the malignant lesions. Barrett’s mucosa above the resected area remained in situ. This procedure generally requires close follow-up, especially in a situation like described in this case where a premalignant lesion has been left in situ. Four-quadrant-biopsy therefore still represents the gold standard for surveillance of BE (Sensitivity 91%) (4).

Since BE is a tissue of high heterogeneity, and four-quadrant-biopsies usually are taken in a mean distance of 2 cm, the risk of unrecognised malignancies through sampling errors is of notable importance (5). Even by using such a systematic biopsy protocol dysplasia and early carcinomas are often grossly occult and can easily be missed. Therefore during esophagectomy, all conspicuous metaplastic tissue besides malignancies should be removed. This was omitted twice during the first operation as well as with the second procedure mucosectomy.

Retrospectively it becomes obvious that in this patient there was a multicentric disease before the first operation since carcinoma and high-grade intraepithelial neoplasia was found within one month at two different locations within the Barrett’s segment. In face of the short amount of time till recurrence, it remains on suspicion, if the dysplastic areas might have been existent and escaped from diagnosis already in February 2005. Consequently, the benefit of local treatment strategies (e.g. mucosectomy) of malignancies of this entity remains highly debatable. A recurrence rate of high-grade intraepithelial neoplasia and early cancer up to 30% in endoscopically treated patients has been reported previously (6).

Other published studies demonstrated multifocality of high-grade intraepithelial neoplasia and/or adenocarcinoma.(7,8) So the presented case is not new in regards of multicentric carcinoma within Barrett’s esophagus, but the diagnosis, management and initial operative approach make it unique and emphasize the need to remove all premalignant tissue in a patient with a long-segment Barrett’s esophagus.

For reconstruction after esophagectomy, the method of using the unmodified stomach for thoracic anastomosis with remnant inconspicuous esophagus is obsolete. Due to the lack of the lower esophageal sphincter, there is a high probability of continuous gastroesophageal reflux and—after a latency period—to develop a BE anew. The preferable technique is to create a gastric tube—by a transhiatal en-bloc esophagectomy and proximal gastrectomy (9). Once a gastric pull-up is used as the reconstruction method it is important to create a small tube and not to pull up the entire stomach, since this will lead to substantial decrease of quality of life for the patient. Other potential reconstruction methods are colonic interposition or in some rare cases jejunal interposition.

Thus, our case underlines impressively the necessity of resection of all Barrett’s mucosa and creating a gastric tube to be pulled upwards in treatment of adenocarcinomas of the esophagus.
